# New magnetostratigraphic evidence for the age of Acheulean tools at the archaeo-palaeontological site “Solana del Zamborino” (Guadix – Baza Basin, S Spain)

**DOI:** 10.1038/s41598-017-14024-5

**Published:** 2017-10-18

**Authors:** C. Álvarez-Posada, J. M. Parés, R. Sala, C. Viseras, S. Pla-Pueyo

**Affiliations:** 1 Geochronology Program, CENIEH, Paseo Sierra de Atapuerca 3, 09002 Burgos, Spain; 2 IPHES (Institut Català de Paleoecologia Humana i Evolució Social). Área de Prehistòria, Universitat Rovira i Virgili, Campus Sescelades-URV, Edifici W3, 43007 Tarragona, Spain; 3 Dpto. Estratigrafía y Paleontología, Facultad de Ciencias, Universidad de Granada, 18071 Granada, Spain; 4 Heriot-Watt University, Edinburgh, EH14 4AS United Kingdom

## Abstract

The sedimentary record in the Guadix-Baza Basin (southern Spain) has proved to be a great source of information for the Miocene through the Pleistocene periods, due to the abundant faunal remains preserved, in some cases associated with lithic tools. The Solana del Zamborino (SZ) section has been the subject of controversy ever since a magnetostratigraphic analysis resulted in an age of 750–770 Kyr for Acheulean tools, a chronology significantly older than the ~600 Kyr established chronology for the first Acheulean record in Europe. Although recent findings at the “Barranc de la Boella” site (north-east of the Iberian Peninsula) seem to indicate that an earlier introduction of such technique in Europe around 0.96–0.781 Ma is possible, the precise age of the classical site at SZ is still controversial. The aim of this paper is to constrain the chronology of the site by developing a longer magnetostratigraphic record. For this purpose, we carried out an exhaustive sampling in a new succession at SZ. Our results provide a ~65 m magnetostratigraphic record in which 4 magnetozones of normal polarity are found. Our new magnetostratigraphic data suggest an age range between 300–480 Kyr for the lithic tools, closer to the age of traditional Acheulean sites in Europe.

## Introduction

The Guadix-Baza basin, located in the Betic Mountain Range (southern Spain) contains the most complete sedimentary record in Spain throughout the Miocene to Pleistocene epochs^1–5^, including well known palaeontological sites such as Orce, Huéscar, Venta Micena, Fonelas, Barranco León and the three sites of Fuente Nueva locality, which have provided abundant faunal information critical for the Plio-Pleistocene boundary^6–12^. Specifically, sites such as Solana del Zamborino (SZ hereafter) have produced abundant lithic tools that have been claimed to be the oldest Acheulean tools in Europe^13^. The aim of this study is to provide a solid and more complete chronological framework for SZ. Our results are based on new magnetostratigraphy that covers approximately over 65 meters of section, complementing the previous results by Scott and Gibert^13^.

Solana del Zamborino is located in the Guadix-Baza Basin (one of the more prominent post-orogenic intramontane depressions of the Betic Range) and developed during the Miocene, with a NE-SW orientation^2,14^. The basin was flooded by sea water during the Tortonian and marine sediments were deposited^15^. Subsequently, the basin was isolated from the sea, due to tectonic tilting related to the activation of a NE-SW fault system, and filled with continental sediments from the late Tortonian to the Upper Pleistocene^16^. During the Villafranchian stage, the basin was divided in two sub-basins separated by a topographic elevation: the Baza sub-basin to the NE and the sub-basin of Guadix to the SW^16^. The Guadix sub-basin holds an abundance of terrigenous sediments, including conglomerates and sands which were deposited by rivers, alluvial fans and lacustrine fan deltas, fed by the surrounding reliefs^17–22^.

**Figure 1:**
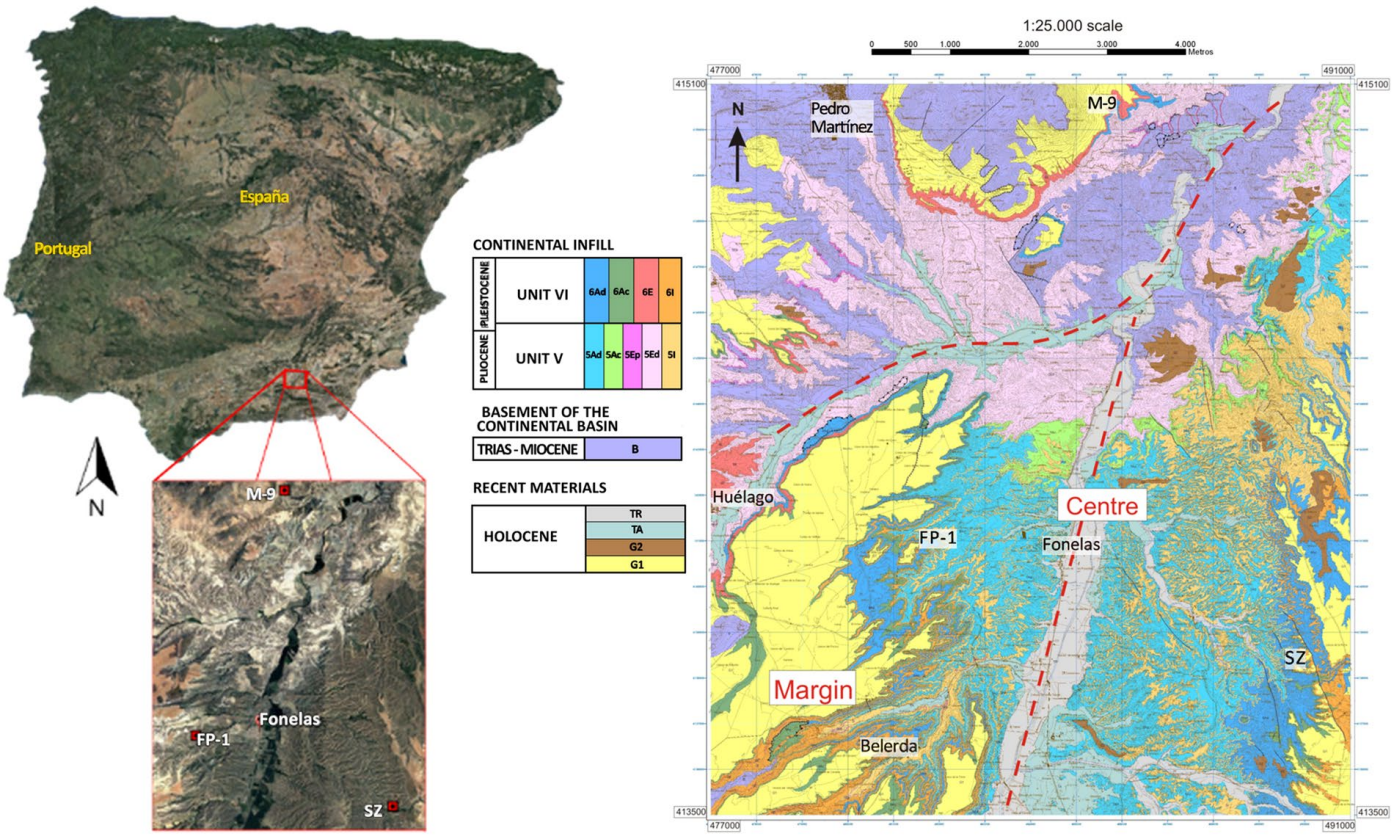
Location of the paleontological sites of Solana del Zamborino (SZ), Mencal (M-9), and Fonelas (FP-1) using Google Earth free program (version 7.1.8.3036. Map data: Google, Digital Globe). The lithostratigraphic map of the Fonelas-Mencal area was created by Pla-Pueyo^20^ and digitalised using ArcGis 9.1. (http://www.esri.com/arcgis/about-arcgis). An online version of the map can be found in http://hdl.handle.net/10481/2373. Legend: **TR**, recent terraces; **TA**, ancient terraces; **G2**, glacis 2; **G1**, glacis 1; **5Ad**, luddites, sands and gravels (locally palustrine carbonates), Detrital Axial System; **5Ac**, palustrine-lacustrine limestones (locally tufa), Axial System carbonates; **5Ep**, blocks and gravels, Proximal External Transverse System; **5Ed**, sands, shales and gravels, distal External Transverse System; **5I**, gravel, sand and shale (locally palustrine-calcrete carbonates), External Transverse System; **6Ad**, sands and shales (locally palustrine-lacustrine carbonates), Detrital Axial System; **6Ac**, palustrine-lacustrine carbonates (locally shale), Carbonate Axial System; **6E**, breccia, gravel, sand and shale, External Transverse System, **6I**, gravel, sand and shale, External Transverse System; **B**, basement.

The site targeted for this study is located on the western sector of the Guadix-Baza Basin, where only the Guadix and Gorafe-Huélago formations are exposed (the two lithostratigraphic units that have survived after the different nomenclatures established over time^23–25^). Deposits are horizontal to sub-horizontal and include frequent lateral facies changes; these fluvial deposits are the two most recent units of the continental landfill of the basin, named as units V and VI^20^ (Fig. 1). The archaeo-paleontological site of SZ is in the eastern sector of the Guadix sub-basin^21^, (Fig. 1), and within the youngest stratigraphic unit (VI, with an age that corresponds to the lower limit of Olduvai subchron, 1.778 My^22^), and south of the highest lacustrine intercalation in Guadix formation as described by Casas *et al*.^24^. Within the site, stratigraphic levels have been traditionally named alphabetically from A to F (from bottom to top) and their detailed description and characteristics are fully included in Casas *et al*.^24^.

**Figure 2:**
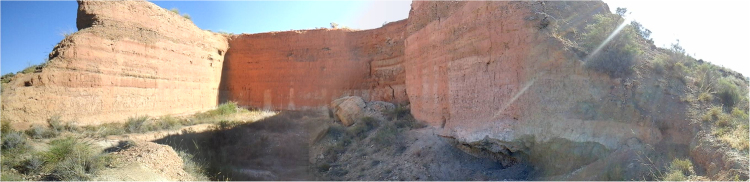
Current state of the paleontological site of Solana del Zamborino. Photograph taken by Claudia Álvarez-Posada. The walls of the quarry are approximately 12 meters in height.

The excavation consists of an open quarry (Fig. 2), in what is known as “Llano de Zamborino” which began as a palaeontological site excavated in 1972 by members of the Department of Prehistory at the University of Granada, led by Botella^26–28^. The quarry has yielded an abundance of skeletal remains and lithic tools, found in an ensemble of layers composed mainly of silts and grey claystone. The stratigraphic, faunal and lithic studies^24,29,30^ have allowed for interpretation of the human occupation of the site, ranging from occasional hunting place to a permanent settlement during periods of hunting and finally to progressive abandonment^26,28,31^.

## Materials and Methods

**Figure 3:**
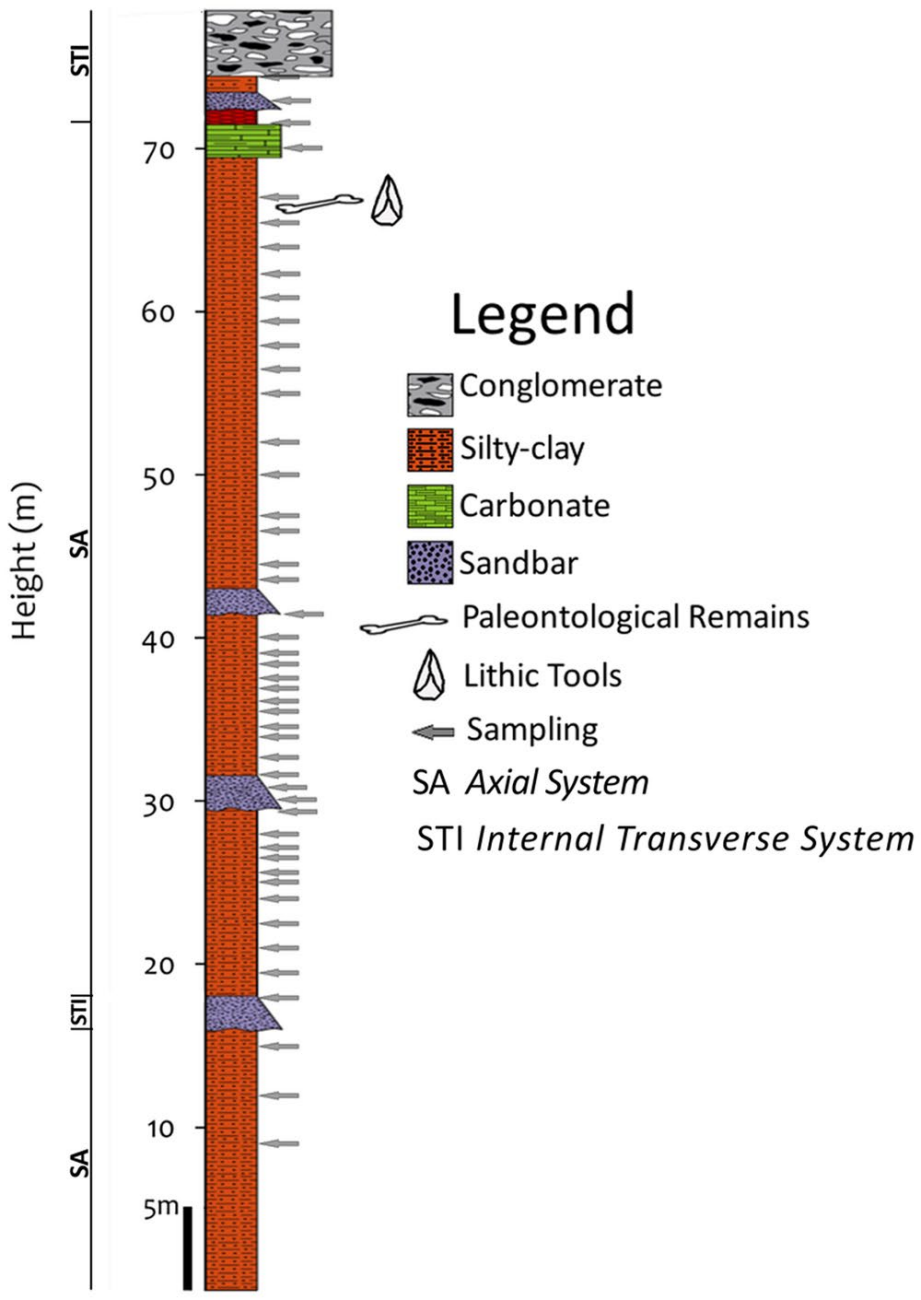
Stratigraphic succession obtained in this study, resulting from combining two parallel sequences measured in SZ in the years 2014 and 2015.

During the years 2014 and 2015, two paleomagnetic campaigns were carried out at the site of Solana del Zamborino, in order to obtain as complete magnetostratigraphic record as possible. For this purpose, a stratigraphic column of the section to be sampled was measured. Since the study area is dissected by several badlands, the process consisted of developing two parallel stratigraphic columns. Because beds are flat lying it has been possible to correlate both sections, which overlap 13.5 meters. Therefore, a composite stratigraphic section of about 80 meters was obtained (Fig. 3). This section is predominantly composed of silty-clay sediments, which have determined the process of sampling. The first sampling technique involved retrieving a compact block using a ceramic knife, while for the second consisted of using a cylindrical non-magnetic device sliced longitudinally into two halves and inserted by hammering into the sediment. In both cases, diluted sodium silicate solution was used to preserve the integrity of the sample. All samples were oriented *in situ* with a standard compass and clinometer.

The SZ section is dominated by green-grey fluvial sediments from the fluvial Axial System. Redder sediments corresponding to several progradations of the Internal Transverse System alluvial fans are intercalated among them. From bottom to almost to the top, the sequence predominantly comprises fine-grained sediments (Fig. 3). Towards the middle part of the section, some coarser-grained beds appear. This lithofacies association can be interpreted as a distal fluvial flood plain, subjected to sporadic periods of ponding and into which sandy sediments come from the overbank of fluvial channels^19–21,32^. The top of the site section is marked by palustrine carbonates, a thin layer of clay, and a bed of conglomerates about two meters thick, related to an important progradation of alluvial fan sediments from the Internal Transverse System into the axial valley.

We sampled every meter and a half or less, whenever possible, in a total of 47 sampling sites from which we have obtained 367 individual samples (between 8 cm^3^ and 11 cm^3^ depending on the process by which the samples were obtained).

The measurement of the natural remanent magnetization (NRM) of the specimens and the progressive demagnetisation was carried out in two different laboratories: a preliminary analysis was carried out in the laboratory of Paleomagnetism and Rock Magnetism at the University of Oxford (England) on a pilot set of specimens. Low field magnetic susceptibility was measured with a Kappabridge model KLY2 with a CS3 oven incorporated. Further analyses were carried out using a cryogenic magnetometer 2 G enterprises DC including an online degausser for alternating field (AF) demagnetization. Subsequently, the remaining specimens were processed at the National Research Center for Human Evolution (CENIEH, Burgos), using an TD-48SC oven (ASC Scientific) for thermal (TH) demagnetization, a cryogenic magnetometer 2 G model 755R-4K with a built in degausser system for AF demagnetization up to 170 mT, and an Impulse Magnetizer, model IM-10–30 (ASC Scientific) for isothermal remanent magnetization (IRM) acquisition curves.

Of the 367 samples obtained, 316 have been analysed, 32 of which have been used to obtain IRM acquisition curves. From the remaining 284 specimens, 175 were analysed by TH demagnetisation with progressive steps of 30^◦^C until 420^◦^C and then increasing the steps to 50 ^◦^C up to 670 ^◦^C; and the last 109 specimens were analysed using AF to a maximum field of 0.1 T. Finally, after visual inspection of the Zijderveld diagrams, the characteristic remanent magnetization (ChRM) direction, the highest stability component of the NRM, was determined and computed for each specimen using Principal Component Analysis^33^, typically anchoring the directions to the origin. We used the Fisherian mean direction^34^ of the ChRM to calculate the corresponding virtual geomagnetic pole (VGP) position for each site (using the software packages Pmag-Tauxe, 1988; VDP7 Ramon & Pueyo, 2014). Latitudes of the VGP poles were used to establish the local magnetostratigraphy.

### Data Availability

The datasets generated during and/or analysed during the current study are available from the corresponding author upon request. The information about the individual samples analysed, and the statistical result are provided as a supplementary information.

## Results

**Figure 4:**
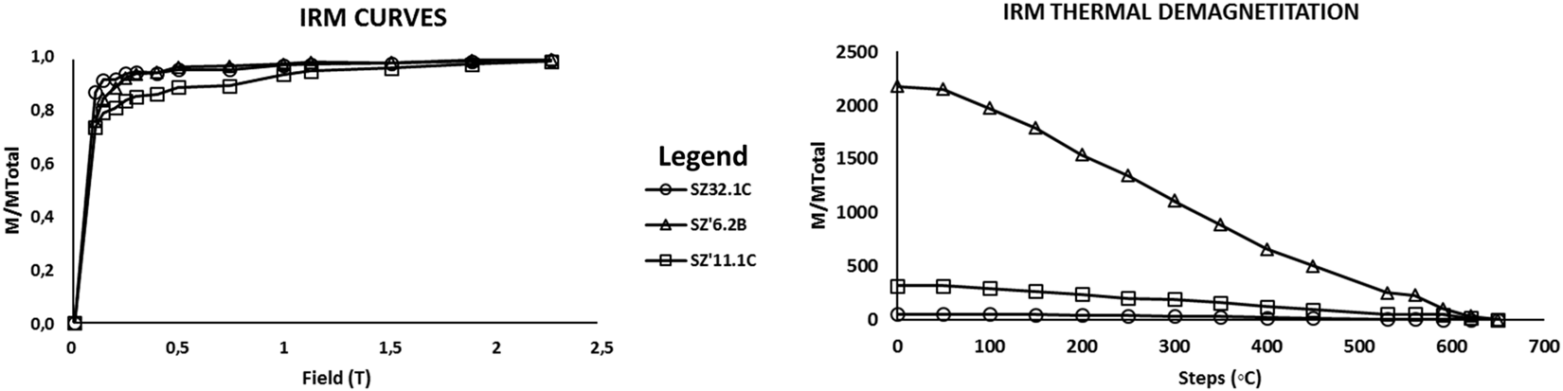
Example of *isothermal remanent magnetization* (IRM) acquisition curves and their respective thermal demagnetisation for some representative specimens. Each symbol corresponds a different sample. Notice a rapid increase in the IRM at fields lower than 1 T (between 300 and 400 mT) and unblocking temperatures just below 600 °C, suggesting magnetite as the main carrier of stable magnetization.

**Figure 5:**
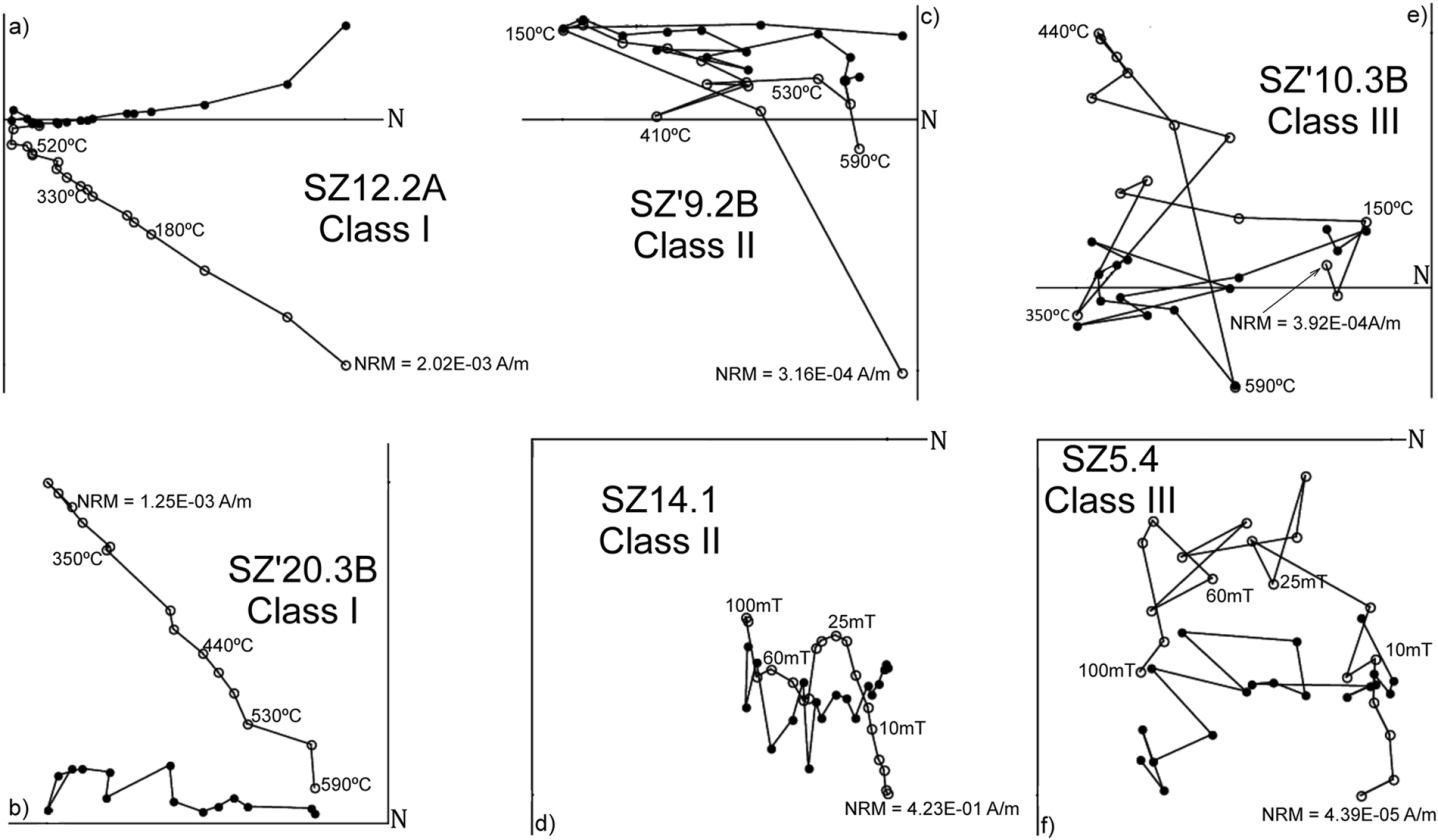
Representative orthogonal demagnetisation diagrams (Zijderveld). Black and white dots represent horizontal and vertical components respectively. Data in geographic coordinates.

The analysed specimens exhibit a wide range of NRM intensities, which vary from 3.02E-05 A/m to a 6.83E-01 A/m, with an average intensity of 6.63E-02 A/m. The IRM acquisition curves indicate relatively low values of coercivity, reaching saturation rapidly towards field values before 1 T (Fig. 4). Thermal demagnetisation of the IRM suggests Curie temperatures close to 625°− 650°C. Such magnetic behaviour (attributed to a mineralogy rich in iron oxides) has been previously observed and reported^20,21^. Overall, the specimens have a stable behaviour during both, AF and TH, demagnetization, and present normal and reverse polarity directions (Fig. 5). Following the visual observation of the Zijderveld diagrams, we identified the presence of a low temperature component (<270 °C) that moves away from the origin, and can be associated with a recent remagnetization. It is followed by a stable component, directed towards the origin, taken as the primary magnetization.

**Figure 6:**
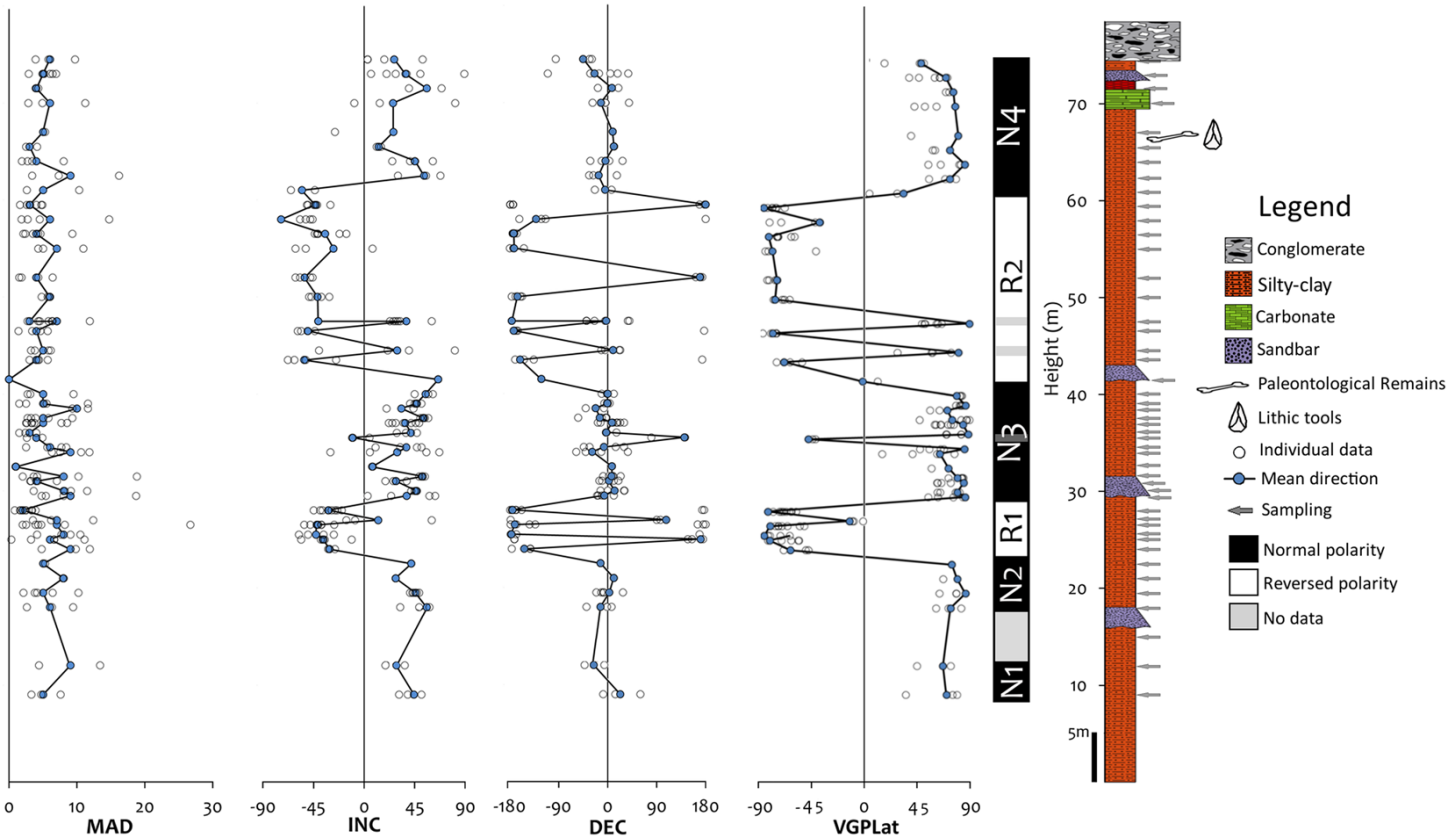
Graphical representation of the virtual geomagnetic pole (VGP) latitude against stratigraphic height, and the resultant local magnetostratigraphy. (MAD: *maximum angular deviation*, Inc/Dec: inclination and declination of each ChRM direction). Four magnetozones with normal polarity, named N1, N2, N3 and N4 and three magnetozones with reversed polarity named R1, R2 have been defined. At least two magnetostratigraphically consecutive sites of the same polarity were required to define a change in polarity, otherwise a grey bar represents a single site.

For the distribution of our samples we proceed by two different methods, a visual inspection at the Zijderveld diagrams about the behaviour of the progressive demagnetisation of each sample, and a statistical distribution of the MAD by calculated mean of all data. In this second method, the value obtained was a mean of 4.7, which we use as reference to classify the data as type I if the MAD value of the individual sample is equal or less at 4.7; and we classified as type II if the MAD is over 4.7. For the samples without possibility to obtain data, the type of such samples has been defined as III. A table with both classifications can be found in the supplementary information (Table 1), and because the difference between them is not so large, we consider maintaining the visual inspection of each Zijderveld diagram of each individual sample. Therefore, samples have been grouped in three different types. Type I (23%) when the ChRM direction is clearly defined with a straight line and pointing towards the origin (Fig. 5a,b); type II (40%) directions are a little bit noisy, but ChRM directions can still be unambiguously defined (Fig. 5c,d); and type III (37%) includes noisy directions that prevent ChRM identification (Fig. 5e,f). We only used those samples in which the primary component could be clearly interpreted, including both type I and type II data, excluding the type III data, to compute mean directions and their corresponding VGP position. Plotting these data against the stratigraphic column produces a local magnetostratigraphy for the Solana del Zamborino site (Fig. 6). There are four magnetozones of normal polarity, labelled N1, N2, N3, and N4 from bottom to top of the sequence, and two reverse magnetozones R1, and R2 in the midsection. Between N1 and N2 levels there is a gap due to the lack of information since it has not been possible to obtain conclusive ChRM directions, therefore we consider such interval as two different magnetozones, but we are aware that a future revision and resampling of that part of the section must be carried out to determine if they are separate magnetozones or not. Nevertheless, to determinate the antipodal nature of the reversal a bootstrap test has been carried out with our data, whose results can be found as the supplementary information (Fig. 1).

**Table 1:** Summary of the published faunal lists of large mammals for Solana del Zamborino (SZ).

Martín Penela (1998) (SZ)	Ruiz-Bustos (1999)
*Macaca sylvanus*	*Macaca sylvanus*
*Mammuthus trogontherii*	*Mammuthus trogontherii*
*Palaeoloxodon antiquus*	*Palaeoloxodon antiquus*
*Equus caballus torralbae*	*Equus caballus*
*Dicerorhinus hemitoechus*	*Stephanorhinus hemitoechus*
*Cervus elaphus*	*Cervus elaphus*
*Capreolus*	*Capreolus*
*Dama sp*.	*Dama sp*.
*Bos (Bos) primigenius*	*Bos primigenius*
*Bos (Bison) priscus*	*Bison priscus*
*Hippopotamus sp*.	*Hippopotamus cf. amphibious*
*Sus scrofa*	*Sus scrofa*
*Canis cf. lupus*	*Canis lupus*
*Panthera (Leo) spelaea*	*Panthera leo spelaea*
*Lynx cf. Pardina*	*Lynx cf. pardina*
*Felis sylvestris*	*Felis sylvestris*

Modified from Jiménez-Arenas *et al*.^39^. More exhaustive information can be found in the bibliography.

**Figure 7:**
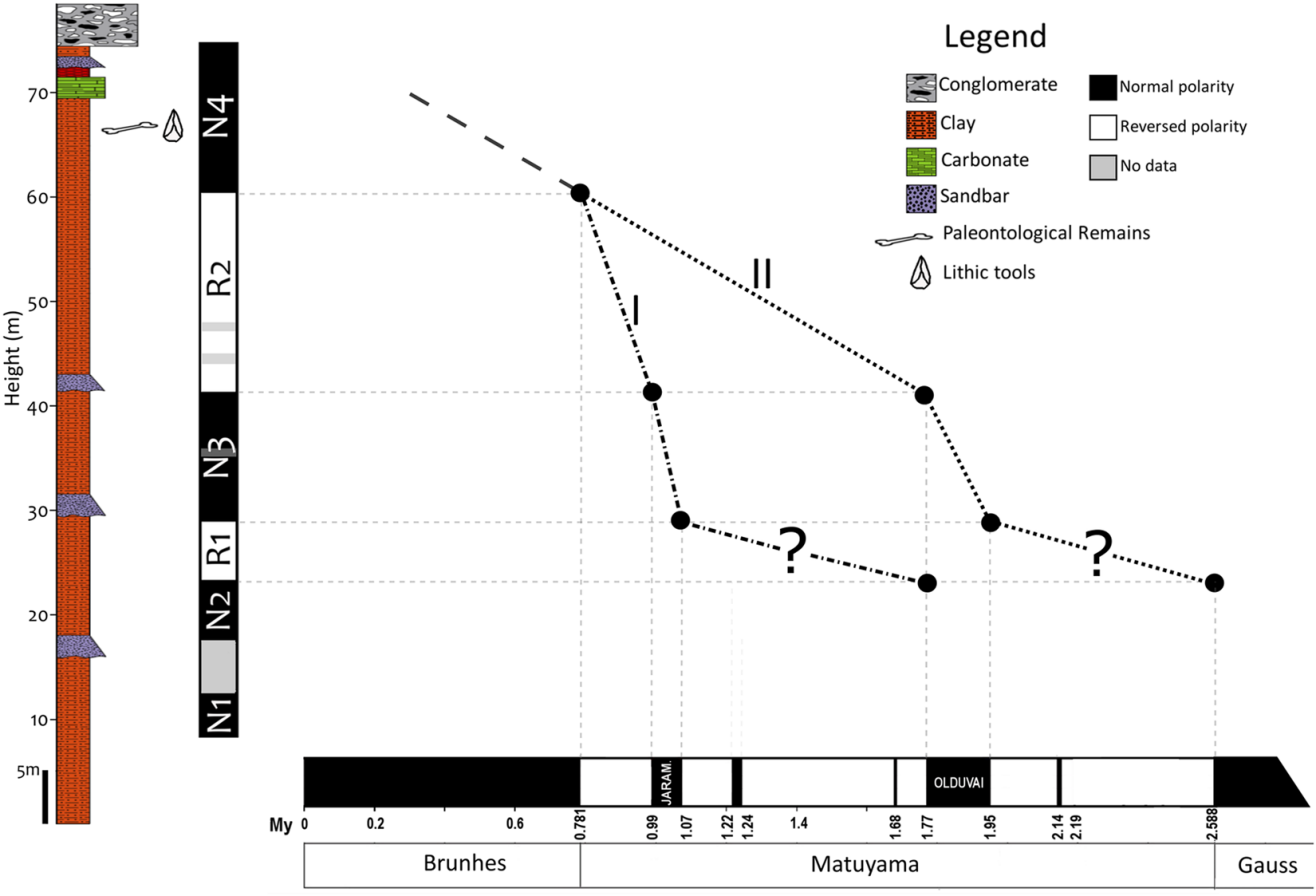
Resultant magnetostratigraphy and suggested correlations to the Global chrono-stratigraphical correlation table for the last 2.7 million years (Cohen and Gibbard^47^). Option II is favoured due to its consistency with the nearby Fonelas magnetostratigraphic section (see text for discussion).

Given the remains of macro vertebrates found at the Solana del Zamborino (Table 1), a chronology of Middle Pleistocene has been traditionally assigned to this site^29,31,35^. Hence, comparing the obtained local magnetostratigraphy to the geomagnetic polarity time scale (GPTS) (Fig. 7), magnetozone N4 found at the top of the section is correlated with the Brunhes Chron (C1n). Therefore, it seems plausible that the next normal magnetozone N3 corresponds to the Jaramillo Subchron, and therefore N2 to the upper limit of Subchron Olduvai. Notice that such correlation (labelled Option I in Fig. 7), implies a rather long Jaramillo Subchron with a concomitant high and variable sedimentation rate. The nearby site of Fonelas (located 5 km away from La Solana, with the Fonelas-Pico 1 (FP-1) section (Fig. 1)) helps in evaluating such correlation. The FP-1 section contains the richest assemblage of vertebrates in the area, which has provided nearly 500 fossil remains that belong to 20 genera of large mammals, as well several remains of small mammals, in which tooth marks by carnivores have been found, without presence of human activity^36,37^. A recent magnetostratigraphy of the section reveals that the Jaramillo Subchron is absent^20–22^. In that locality, the stratigraphic record begins at the top of Chron Gauss and finishes in the Brunhes Chron. Therefore, and given the proximity to the SZ section, it seems likely that the Jaramillo could be missing as well in La Solana. Under such assumption, an alternative option (Option II in Fig. 7) includes assigning N3 magnetozone to Olduvai Subchron, and N2 magnetozone to the upper limit of Gauss Chron (C2n).

**Figure 8:**
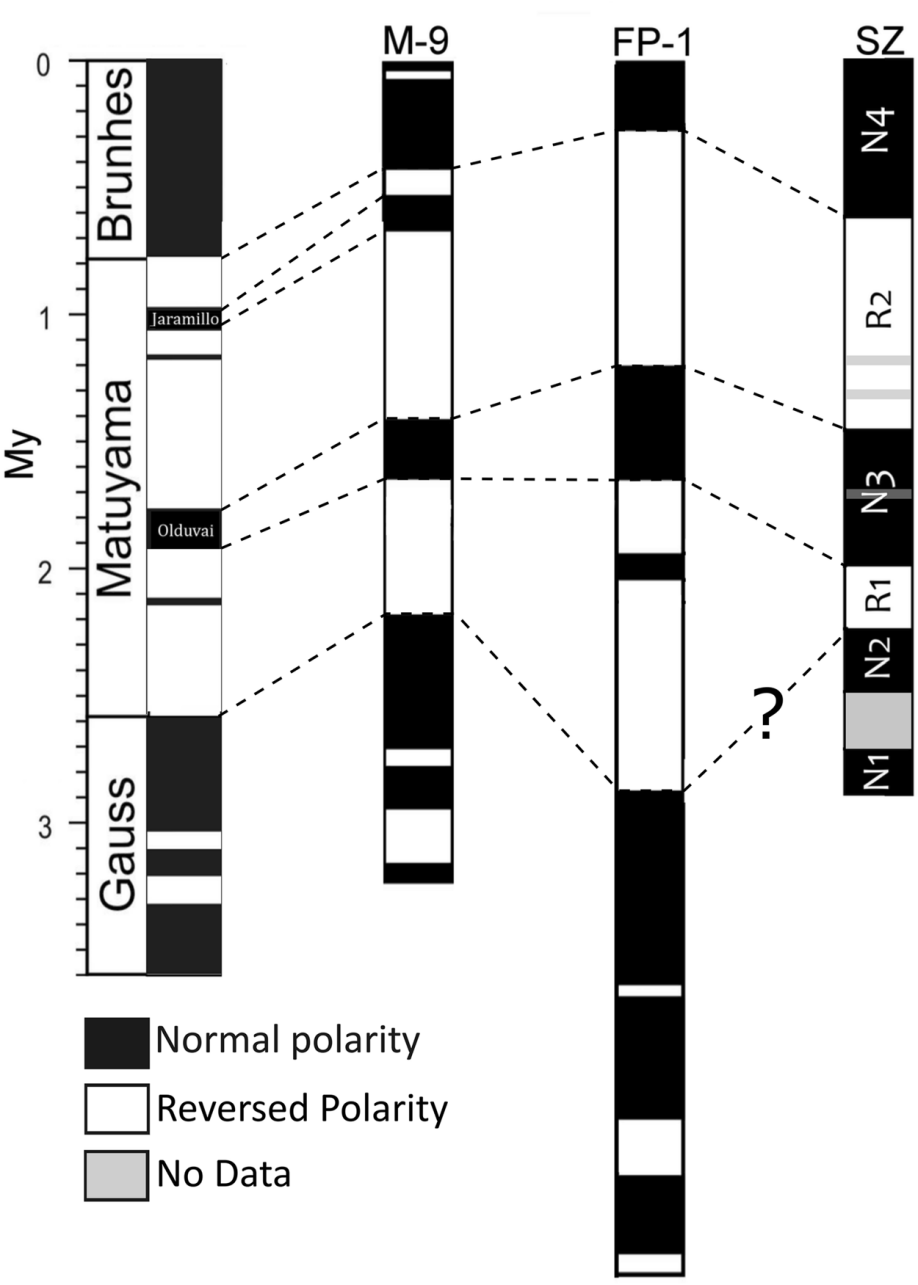
Correlation between Mencal (M9), Fonelas (FP-1) and Solana del Zamborino (SZ) magnetostratigraphies and the Geomagnetic Polarity Time Scale (GPTS 2004^48^) modified from Pla-Pueyo^20^.

In support of such interpretation are the bulk sedimentation rates estimated in La Solana, Fonelas and Mencal sites (the last one located at 14 km N form Solana, with the M-9 section^20^), which are of the same order of magnitude (average of 2 cm/kyr^20–22^) (Fig. 8).

## Discussion

The ultimate goal of establishing the chronology at SZ resides in the presence of the lithic tools found at a stratigraphic level reported as level B, corresponding to the Guadix Formation (more information can be found in Casas *et al*.^24^ and Botella *et al*.^27^). In a previous study^13^ the archaeological layer was found above the Brunhes / Matuyama boundary. Based on an inferred accumulation rate, an age of ~760kyr was suggested by these authors. However, because this previous magnetostratigraphy lacks polarity reversals below and above the archaeological layer, an alternative stratigraphic section at the paleontological site of Cúllar-Baza^38^ (located more than 50 km away), was used to infer the accumulation rate for La Solana. We would like to emphasize that the section of Cúllar-Baza is not lithologicaly similar to that at La Solana, since the latter is characterised by siliciclastic sediments (silt and clay of fluvial origin), whereas the former sequence is mainly composed of carbonate lacustrine-palustrine sediments^5^. Highly different sediment accumulation rates are expected to be found simply because of the contrasting depositional environment. Jimenez-Arenas *et al*.^39^ have also brought up inconsistencies with such an age assignment. According to these authors, there are several errors in the interpretation of the archaeological assemblage for the site, and also discrepancies with the original faunal lists published. In our new study, we have been able to locate three reversals below the archaeological site at La Solana, which allows a firmer interpretation of the local magnetostratigraphy and by inference the approximate chronology of the site. Assuming an average accumulation rate of 2 cm/kyr throughout La Solana section, the stratigraphic position of the archaeological site would have an estimated age range between 300 Kyr to 480 Kyr, significantly younger than the previously suggested age, and more in line with the conventional chronology proposed for the evolution of Acheulean in Europe^40^ and with the faunal record of the SZ site itself^31^.

## Conclusions

An exhaustive paleomagnetic study of the tool-bearing and paleontological deposits at La Solana del Zamborino, as well as of the older stratigraphic levels, has been carried out:

- A stratigraphic sequence of ~65 meters has been paleomagnetically studied.
- The samples generally present a stable magnetic signal and quality that have allowed us to obtain an interpretable magnetostratigraphy.
- The absence of subchron Jaramillo is consistent with the existing records obtained by Pla-Pueyo^20^and Pla-Pueyo *et al*.^21^ in the close palaeontological site of Fonelas P-1.

This new magnetostratigraphic study, based on three polarity reversals, is consistent with existing data from nearby sections (e.g., FP-1 and M-9) and based on the sedimentation rate, the stratigraphic position of the archaeological site at SZ would have an approximate age of ~300–480 Kyr, much younger than the age provided by Scott and Gibbert^1^ and, therefore, more similar to the chronology of European Acheulean sites such as L’Arago in France, Boxgrove in England or Atapuerca in Spain^41–46^.

## Electronic supplementary material

Supplementary Information
