# Supplementary material for: New magnetostratigraphic evidence for the age of Acheulean tools at the archaeo-palaeontological site “Solana del Zamborino” (Guadix – Baza Basin, S Spain)
Source: Sci Rep. 2017 Oct 18;7:13495. doi: 10.1038/s41598-017-14024-5 (PMC5647401; doi:10.1038/s41598-017-14024-5)

Supplementary Information.

New magnetostratigraphic evidence for the age of Acheulean tools at the archaeo-palaeontological site "Solana del Zamborino" (Guadix – Baza Basin, S Spain)

C. Álvarez-Posada[1*]; J.M. Parés[1]; R. Sala[2]; C. Viseras[3]; S. Pla-Pueyo[4]

[1]Geochronology Program, CENIEH, Paseo Sierra de Atapuerca 3, 09002-Burgos, Spain
[2]IPHES (Institut Català de Paleoecologia Humana i Evolució Social). Área de Prehistòria, Universitat Rovira i Virgili, Campus Sescelades-URV, Edifici W3. 43007 Tarragona
[3]Dpto. Estratigrafía y Paleontología, Facultad de Ciencias, Universidad de Granada. 18071, Granada, Spain.
[4]Heriot-Watt University, Edinburgh. EH14 4AS United Kingdom
* Corresponding author. E-mail address: claudiaalvarezposada@gmail.com

This supplementary information contains one table and one figure.

Table 1

Summary of the paleomagnetic data.

Height (m): Stratigraphic height of the sequence beginning from the bottom,. SZ sites are those from the sequence carried out in 2014, and SZ' are those sampling at the sequence carried out in 2015. **Analysis:** paleomagnetic demagnetization and analysis carried out for each sample (**TH** thermal demagnetization; **AF**, demagnetization by alternating fields; and **IRM** the isothermal remanent magnetization curves). **ChRM Directions:** Characteristic Remanent Magnetization direction of each sample; **Dec/Inc are** the declination/Inclination of each individual sample; and **MAD** is the maximum angular deviation. **Type: visual**, is the visual inspection of the Zijderveld diagrams of each sample and their behaviour during the demagnetization, grouped the samples in three different types, type I (23%), type II (40%) and type III (35%); **MAD**, in this distribution we have calculated first the mean of the MAD of all the individual samples, obtained a value of 4.7 which we use as reference to classify the data as type I if the MAD value of the individual sample is ≤4.7; and we classified as type II if the MAD is > 4.7. For the samples without possibility to obtain data the type has been defined as III. **N:** number of data points. **Dec:** mean declination of the sampling site. **Inc:** mean inclination of the sampling site. ***K***: the precision parameter. **Class:** statistical classification of the data by using the precision parameter, *k*, in which *k*≥10 = class I, *k<10* = class II, and when just a one sample has been used to calculate the mean data, *k* = class 3. **Watson's_f:** is the Watson's test for randomness, where **F stats** is the Watson's F statistics of the directional data of each sampling site, and the next column named as **Beat**, is the number to beat for the F stats; if the first columns has a value greater than the required number to beat, the data failed to pass the test and are randomly distributed.

Field Data				Analysis			ChRM directions			Sample Type		Site Mean Distribution					Watsons_f	
Height (m)	Sampling Site	Individual Sample	Dec / Inc	TH	AF	IRM	Dec	Inc	MAD	Visual	MAD	N	Dec	Inc	k	Class	F stats	Beat
		SZ'1.1A	020/70	X			352	39	4,7	I	II							
		SZ'1.1B	020/70	x			-	-	-	III	III							
		SZ'1.2A	023/74	x			-	-	-	III	III							
9	SZ'1	SZ'1.2B	023/74	X			14	51	3,3	I	I	3	23,5	44,5	8,1	2	0	4,46
		SZ'1.2C	023/74		X		60	31	7,6	II	II							
		SZ'1.3	088/63	x			-	-	-	III	III							
		SZ'1.4	045/66			x												
		SZ'2.1A	103/61	X			-	-	-	III	III							
		SZ'2.1B	103/61		X		-	-	-	III	III							
10,5	SZ'2	SZ'2.1C	103/61	x			-	-	-	III	III							
		SZ'2.2A	131/52			x												
		SZ'2.3A	106/55	x			-	-	-	III	III							
		SZ'2.3B	106/55	X			-	-	-	III	III							
		SZ'3.1A	092/22	x			-	-	-	III	III							
		SZ'3.1B	092/22			x												
		SZ'3.1C	092/22		X		354	36	4,4	I	I							
12	SZ'3	SZ'3.2A	125/32		X		-	-	-	III	III	2	334.6	28.7	10.3	1	0	4,46
		SZ'3.2B	125/32	x			-	-	-	III	III							
		SZ'3.3A	123/55	X			318	19	13,4	I	II							
		SZ'4.1A	0/0	X			-	-	-	III	III							
		SZ'4.1B	0/0	x			-	-	-	III	III							
		SZ'4.1C	0/0			x												
13,5	SZ'4	SZ'4.1D	0/0		X		-	-	-	III	III							
		SZ'4.2A	0/0	x			-	-	-	III	III							
		SZ'4.2B	0/0	X			-	-	-	III	III							
		SZ'4.2C	0/0		X		-	-	-	III	III							
		SZ'5.1A	358/70	x			-	-	-	III	III							
		SZ'5.1B	358/70			x												
		SZ'5.1C	358/70	X			-	-	-	III	III							
		SZ'5.2A	332/70	X			-	-	-	III	III							
15	SZ'5	SZ'5.2B	332/70	x			-	-	-	III	III							
		SZ'5.2C	332/70		X		-	-	-	III	III							
		SZ'5.3A	286/38	x			-	-	-	III	III							
		SZ'5.3B	286/38	X			-	-	-	III	III							
		SZ'5.3C	286/38		X		-	-	-	III	III							
		SZ'5bisA	0/0	x			-	-	-	III	III							
		SZ'6.1A	244/53	x			-	-	-	III	III							
		SZ'6.1B	244/53			x												
18	SZ'6	SZ'6.2A	0/0	X			7	49	6,3	II	II	3	352	47,9	#####	1	0	4,5
		SZ'6.2B	0/0		X		321	58	9,4	II	II							
		SZ'6.2C	0/0		x		359	32	9	II	II							

Field Data				Analysis			ChRM directions			Sample Type		Site Mean Distribution					Watsons_f	
Height (m)	Sampling Site	Individual Sample	Dec / Inc	TH	AF	IRM	Dec	Inc	MAD	Visual	MAD	N	Dec	Inc	k	Class	F stats	Beat
		SZ'7.1A	281/52	x			-	-	-	III	III							
		SZ'7.1B	281/52		X		352	44	3,8	II	I							
		SZ'7.2A	332/52	x			-	-	-	III	III							
19,5	SZ'7	SZ'7.2B	332/52	X			348	47	6,4	II	II	3	3,1	45,5	25,4	1	0	4,46
		SZ'7.2C	332/52			x												
		SZ'7.3A	332/52	x			-	-	-	III	III							
		SZ'7.3B	034/79	X			28	41	10,2	II	II							
		SZ'8.1A	0/0	x			-	-	-	III	III							
		SZ'8.1B	0/0	X			-	-	-	III	III							
22,5	SZ'8	SZ'8.1C	0/0		X		11	28	7,9	II	II	1	11	28	-	3	-	-
		SZ'8.2A	0/0	x			-	-	-	III	III							
		SZ'8.2B	0/0	X			-	-	-	III	III							
		SZ'9.2A	0/0	x			-	-	-	III	II							
		SZ'9.2B	0/0	X			218	-26	4,8	II	II							
		SZ'9.2C	0/0		X		-	-	-	III	III							
24	SZ'9	SZ'9.3A	0/0	x			-	-	-	III	III	3	207	-30,6	22,4	1	0	4,46
		SZ'9.4A	0/0	x			-	-	-	III	III							
		SZ'9.4B	0/0	X			184	-32	9,8	II	II							
		SZ'9.4C	0/0		X		218	-31	11,9	II	II							
		SZ9.1A	0 / 0	x			189	-56	11,1	II	II							
		SZ9.1B	0 / 0		X		155	-38,5	3,2	II	I							
25	SZ9	SZ9.2A	0 / 0	X			189	-11	0,3	II	I	5	170,8	-36,5	13,4	1	0	3,63
		SZ9.2B	0 / 0		X		147	-36	6,6	I	II							
		SZ9.2C	0 / 0		X		175	-35	6,6	II	II							
		SZ9.3	0 / 0	X			-	-	-	III	III							
		SZ'10.1A	0/0	x			-	-	-	III	III							
		SZ'10.1B	0/0	X			183	-50	10,5	II	II							
		SZ'10.1C	0/0		X		169	-58	4,1	II	I							
		SZ'10.1D	0/0			x												
		SZ'10.2A	0/0	x			-	-	-	III	III							
25,5	SZ'10	SZ'10.2B	0/0	X			182	-36	9	II	II	4	182,9	-43	27,9	1	0	3,89
		SZ'10.2C	0/0		X		-	-	-	III	III							
		SZ'10.3A	0/0	x			-	-	-	III	III							
		SZ'10.3B	0/0	X			-	-	-	III	III							
		SZ'10.3C	0/0		X		192	-27	7,7	II	II							

Field Data				Analysis			ChRM directions			Sample Type		Site Mean Distribution					Watsons_f	
Height (m)	Sampling Site	Individual Sample	Dec / Inc	TH	AF	IRM	Dec	Inc	MAD	Visual	MAD	N	Dec	Inc	k	Class	F stats	Beat
26,5	SZ10	SZ10.1A	0 / 0	x			226,9	-53,2	26,7	II	II	8	187,9	-41	21,6	1	0	3,34
		SZ10.1B	0 / 0		X		180	-38	3,9	II	I							
		SZ10.1C	0 / 0			x												
		SZ10.2A	0 / 0	X			182	-38	4,7	I	II							
		SZ10.2B	0 / 0		X		176	-26,8	2,5	II	I							
		SZ10.2C	0 / 0		x		180	-30	5	I	II							
		SZ10.3A	0 / 0	x			-	-	-	III	III							
		SZ10.3B	0 / 0		x		165	-41	8,2	I	II							
		SZ10.3C	0 / 0		X		192,4	-47,8	2	II	I							
		SZ10.3D	0 / 0	x			216,5	-41,3	4,3	II	I							
27	SZ'11	SZ'11.1A	0/0	x			-	-	-	III	III	5	95,5	-1,5	3,5	2	0	3,63
		SZ'11.1B	0/0	X			97	-16	12,4	II	II							
		SZ'11.1C	0/0		X		82	-20	8,8	II	II							
		SZ'11.2A	0/0	x			-	-	-	III	III							
		SZ'11.2B	0/0	X			90	-8	3,5	I	I							
		SZ'11.2C	0/0		X		83	-12	4,2	II	I							
		SZ'11.3A	0/0	x			-	-	-	III	III							
		SZ'11.3B	0/0	X			182	60	6,2	II	II							
		SZ'11.3C	0/0			x												
28	SZ11	SZ11.1A	0 / 0	x			190	-24	1,6	I	I	9	184,3	-32,8	49,3	1	0	3,29
		SZ11.1B	0 / 0		X		181	-32	3,9	I	I							
		SZ11.1C	0 / 0			x												
		SZ11.2A	0 / 0	X			192	-39	2,5	I	I							
		SZ11.2B	0 / 0		X		183,7	-37,1	0,8	I	I							
		SZ11.2C	0 / 0	X			185	-45	1,7	II	I							
		SZ11.3	0 / 0		X		202	-26	3,4	II	I							
		SZ11.4A	0 / 0	x			177	-20	2,3	II	I							
		SZ11.4B	0 / 0		X		174	-29	3,3	II	I							
		SZ11.4C	0 / 0		x		173	-40	2,8	I	I							
29,5	SZ12	SZ12.1	0 / 0	x			-	-	-	III	III	4	12,5	46,6	27,4	1	0	3,89
		SZ12.2A	0 / 0		X		350	58	4,8	I	II							
		SZ12.2B	0 / 0	X			341	63	5,4	I	II							
		SZ12.3A	0 / 0	x			343	24	8	II	II							
		SZ12.3B	0 / 0		x		11	3	18,7	II	II							
30	SZ'12	SZ'12.1A	0/0	x			-	-	-	III	III	4	9	53,9	19,6	1	0	3,89
		SZ'12.1B	0/0	X			360	45	3,6	II	I							
		SZ'12.1C	0/0		X		30	46	9,1	II	II							
		SZ'12.2A	0/0	x			-	-	-	III	III							
		SZ'12.2B	0/0	X			-	-	-	III	III							
		SZ'12.2C	0/0			x												
		SZ'12.3A	0/0	x			-	-	-	III	III							
		SZ'12.3B	0/0	X			347	47	11,5	II	II							
		SZ'12.3C	0/0		X		31	42	8,2	II	II							

Field Data				Analysis			ChRM directions			Sample Type		Site Mean Distribution					Watsons_f	
Height (m)	Sampling Site	Individual Sample	Dec / Inc	TH	AF	IRM	Dec	Inc	MAD	Visual	MAD	N	Dec	Inc	k	Class	F stats	Beat
		SZ13.1A	0 / 0	x			20	26	4,2	I	I							
		SZ13.1B	0 / 0		X		345	28	7	I	II							
31	SZ13	SZ13.1C	0 / 0		x		22	35	5	I	II	5	5,9	30,1	24,1	1		
		SZ13.2A	0 / 0	X			350	38	3,1	I	I						0	3,63
		SZ13.2B	0 / 0		X		11	19	3,2	I	I							
		SZ'13.1A	0/0	x			-	-	-	III	III							
		SZ'13.1B	0/0	X			10	54	3,6	II	I							
		SZ'13.1C	0/0		X		352	51	2	II	I							
		SZ'13.2A	0/0	x			-	-	-	III	III	5	7,4	52,1	116,3	1		
31,5	SZ'13	SZ'13.2B	0/0	X			2	49	4,1	II	I							
		SZ'13.2C	0/0		X		24	52	10,2	II	II						0	3,63
		SZ'13.3A	0/0	x			-	-	-	III	III							
		SZ'13.3B	0/0	X			10	52	18,8	II	II							
		SZ'13.3C	0/0			x												
32,5	SZ14	SZ14.1	0 / 0	x			-	-	-	III	III	1	8	8	-	3		
		SZ14.2	0 / 0		x		10	12	2	II	I							
		SZ15.1A	0 / 0	x			348	67	6,3	II	II							
		SZ15.1B	0 / 0		X		302	-30	7,5	II	II							
		SZ15.2A	0 / 0	X			-	-	-	III	III							
34	SZ15	SZ15.2B	0 / 0		X		320	5	10,6	II	II	4	331,7	29,5	2,3	2	0	3,89
		SZ15.2C	0 / 0			x					I							
		SZ15.3A	0 / 0	X			-	-	-	III	III							
		SZ15.3B	0 / 0		X		37	55,3	11,8	II	II							
		SZ'14.1A	0/0	x			-	-	-	III	III							
		SZ'14.1B	0/0	X			15	32	5,6	II	II							
		SZ'14.1C	0/0		X		316	51	2,5	II	I							
		SZ'14.2A	0/0	x			-	-	-	III	III							
34,5	SZ'14	SZ'14.2B	0/0	X			30	47	7,7	II	II	5	353,1	37,6	7,9	2	0	3,63
		SZ'14.2C	0/0		X		327	10	8,4	II	II							
		SZ'14.3A	0/0	x			-	-	-	III	III							
		SZ'14.3B	0/0	X			359	32	2,6	II	I							
		SZ'14.3C	0/0			x												
		SZ16.1A	0 / 0	x			158	4,9	5	II	II							
35,5	SZ16	SZ16.1B	0 / 0		X		142	-11	3	II	I	3	154,4	-0,3	32,1	1	0	4,46
		SZ16.1C	0 / 0		X		-	-	-	III	III							
		SZ16.2	0 / 0		x		163	6	4,7	II	I							

Field Data				Analysis			ChRM directions			Sample Type		Site Mean Distribution					Watsons_f	
Height (m)	Sampling Site	Individual Sample	Dec / Inc	TH	AF	IRM	Dec	Inc	MAD	Visual	MAD	N	Dec	Inc	k	Class	F stats	Beat
		SZ'15.1A	0/0	x			-	-	-	III	III							
		SZ'15.1B	0/0		X		357	47	1,5	I	I							
		SZ'15.1C	0/0			x												
		SZ'15.2A	0/0	x			-	-	-	III	III							
36	SZ'15	SZ'15.2B	0/0	X			16	37	2,4	I	I	5	357,1	41,6	39,6	1	0	3,63
		SZ'15.2C	0/0	x			358	29	4	I	I							
		SZ'15.3A	0/0	x			-	-	-	III	III							
		SZ'15.3B	0/0	X			353	49	4,1	II	I							
		SZ'15.3C	0/0		X		339	43	2,1	II	I							
		SZ17.1A	0 / 0	x			30	35	3,7	II	I							
		SZ17.1B	0 / 0		X		347	25	3,4	II	I							
		SZ17.1C	0 / 0		X		342	22	8,5	I	II							
37	SZ17	SZ17.1D	0 / 0	X			16	28	2,6	II	I	7	8,2	36,5	19,3	1	0	3,4
		SZ17.3A	0 / 0	X			23	49	3,3	II	I							
		SZ17.3B	0 / 0		X		18	46	2,5	II	I							
		SZ17.4	0 / 0		X		10	42	7,7	II	II							
		SZ'16.1A	0/0	x			-	-	-	III	III							
		SZ'16.1B	0/0	X			357	53	3,9	I	I							
		SZ'16.1C	0/0		X		358	52	3,2	I	I							
		SZ'16.2A	0/0	x			-	-	-	III	III							
37,5	SZ'16	SZ'16.2B	0/0	X			349	54	5,8	I	II	6	345,6	52,8	37,3	1	0	3,49
		SZ'16.2C	0/0		X		357	57	2,7	I	I							
		SZ'16.3A	0/0	x			-	-	-	III	III							
		SZ'16.3B	0/0	X			306	50	9,3	I	I							
		SZ'16.3C	0/0		X		347	42	5,3	II	I							
		SZ'16.3D	0/0			x												
		SZ18.1A	0 / 0	x			-	-	-	III	III							
38,5	SZ18	SZ18.1B	0 / 0		X		320	44	11,6	II	II	2	338.8	33,1	10	1	0	6,94
		SZ18.2	0 / 0		x		353	20	9,3	II	II							
		SZ'17.1A	0/0	x			-	-	-	III	III							
		SZ'17.1B	0/0	X			4	47	11,6	I	II							
		SZ'17.1C	0/0			x												
		SZ'17.2A	0/0	X			0	47	1,5	I	I							
39	SZ'17	SZ'17.2B	0/0		X		360	45	5,5	I	II	5	359,5	46,2	346,2	1	0	3,63
		SZ'17.2C	0/0	x			-	-	-	III	III							
		SZ'17.3A	0/0	x			-	-	-	III	III							
		SZ'17.3B	0/0	X			354	51	2,5	I	I							
		SZ'17.3C	0/0		X		359	41	5,2	I	II							
		SZ19.1A	0 / 0	x			359	45	9,5	II	II							
40	SZ19	SZ19.1B	0 / 0		X		349	61	3,1	II	I	3	360	54,6	62,2	1	0	4,46
		SZ19.2	0 / 0		X		11	57	2,7	II	I							

Field Data				Analysis			ChRM directions			Sample Type		Site Mean Distribution					Watsons_f	
Height (m)	Sampling Site	Individual Sample	Dec / Inc	TH	AF	IRM	Dec	Inc	MAD	Visual	MAD	N	Dec	Inc	k	Class	F stats	Beat
41,5	SZ20	SZ20.1	0 / 0	x			238	66	3.2	II	I	2	239,8	68	720,5	1	0	6,94
		SZ20.2	0 / 0		x		242	70	3.8	II	I							
43,5	SZ'18	SZ18'.1A	0/0	x			-	-	-	III	III	4	199,4	-53,2	13,1	1	0	3,89
		SZ18'.1B	0/0	X			-	-	-	III	III							
		SZ18'.1C	0/0		X		224	-52	4,4	II	I							
		SZ18'.2A	0/0	x			-	-	-	III	III							
		SZ18'.2B	0/0	X			206	-62	3	II	I							
		SZ18'.2C	0/0		X		190	-25	5,7	II	II							
		SZ18'.3A	0/0	x			-	-	-	III	III							
		SZ18'.3B	0/0	X			174	-68	4,2	II	I							
		SZ18'.3C	0/0		X		-	-	-	III	III							
44,5	SZ'19	SZ18'.1D	0/1			x						4	10,7	28,1	2,6	2	0	3,89
		SZ22.1A	0 / 0	x			355,2	80,6	3,8	II	I							
		SZ22.1B	0 / 0		X		21	-40	6,1	II	II							
		SZ22.2A	0 / 0	X			348	40	5,8	II	II							
		SZ22.2B	0 / 0		X		-	-	-	III	III							
		SZ22.2C	0 / 0	X			-	-	-	III	III							
		SZ22.2D	0 / 0		X		23	21	3,2	II	I							
45	SZ'19	SZ'19.1A	306/52	x			-	-	-	III	III							
		SZ'19.1B	306/52	X			-	-	-	III	III							
		SZ'19.2A	004/65		x													
		SZ'19.2B	004/65	x			-	-	-	III	III							
		SZ'19.3A	323/77	x			-	-	-	III	III							
		SZ'19.3B	323/77	X			-	-	-	III	III							
46,5	SZ'20	SZ'20.1A	348/28	x			-	-	-	III	III	5	187,4	-50,2	84,8	1	0	3,63
		SZ'20.1B	348/28	X			192	-59	4,6	I	I							
		SZ'20.1C	348/28			x												
		SZ'20.2A	0/0	x			-	-	-	III	III							
		SZ'20.2B	0/0	X			195	-50	5,7	I	II							
		SZ'20.2C	0/0		X		177	-56	1,4	I	I							
		SZ'20.3A	0/0	x			-	-	-	III	III							
		SZ'20.3B	0/0	X			189	-44	3,4	I	I							
		SZ'20.3C	0/0		X		184	-41	2,7	I	I							
47,5	SZ24	SZ24.1A	0 / 0	x			321	60	4,3	II	I	6	57,6	37,6	6,6	2	0	3,49
		SZ24.1B	0 / 0		X		355	23	11,9	I	II							
		SZ24.1C	0 / 0			x												
		SZ24.2	0 / 0	X			-	-	-	III	III							
		SZ24.3A	0 / 0	X			40	26	6,4	II	II							
		SZ24.3B	0 / 0		X		37	28	4,5	II	I							
		SZ24.4A	0 / 0	X			321	30	5,8	II	II							
		SZ24.4B	0 / 0		X		336	32	6,3	I	II							

Field Data				Analysis			ChRM directions			Sample Type		Site Mean Distribution					Watsons_f	
Height (m)	Sampling Site	Individual Sample	Dec / Inc	TH	AF	IRM	Dec	Inc	MAD	Visual	MAD	N	Dec	Inc	k	Class	F stats	Beat
		SZ26.1A	0 / 0	x			191	-49	6	II	II							
		SZ26.1B	0 / 0		X		184	-31	5,7	II	II							
		SZ26.1D	0 / 0			x												
50	SZ26	SZ26.2A	0 / 0	X			200	-47	4,8	II	II	4	194,2	-41,5	57,4	1	0	3,89
		SZ26.2B	0 / 0		X		-	-	-	III	III							
		SZ26.3A	0 / 0	X			203	-38	5,9	I	II							
		SZ26.3B	0 / 0		X		-	-	-	III	III							
		SZ27.1A	0/0	x			-	-	-	III	III							
		SZ27.1B	0/0	X			170	-46	4,3	II	I							
		SZ27.1C	0/0		X		165	-48	1,5	II	I							
52	SZ'27	SZ27.2A	0/0	x			-	-	-	III	III	4	169,4	-53	117,2	1	0	3,89
		SZ27.3A	0/0	x			-	-	-	III	III							
		SZ27.3B	0/0	X			170	-57	6,4	II	II							
		SZ27.3C	0/0		X		174	-61	1,8	II	I							
		SZ29.1A	150 / 4	x			206	7,4	10,9	II	II							
		SZ29.1A	0/0	x			-	-	-	III	III							
		SZ29.1B	150 / 4		X		187	-52	4	II	I							
55	SZ29	SZ29.1C	0/0		X		-	-	-	III	III	5	232,7	-56	2,5	2	0	3,63
		SZ29.2A	0 / 10	X			336	-42	12,8	II	II							
		SZ29.2B	0 / 10		X		293	-46	5,9	II	II							
		SZ29.2C	0/10	X			187	-52	4,3	II	I							
		SZ29.2D	0 / 10			x												
		SZ30.1A	0 / 0	x			193	-44	3,5	I	I							
		SZ30.1B	0 / 0		X		188	-42	9,3	II	II							
		SZ30.1C	0 / 0			x												
56,5	SZ30	SZ30.2A	0 / 0	X			187	-22	2,4	I	I	6	187,7	-34,6	42,9	1	0	3,49
		SZ30.2B	0 / 0		X		185	-16	4,3	II	I							
		SZ30.3A	0 / 0	X			189	-42	2,1	I	I							
		SZ30.3B	0 / 0		X		186	-41	4,6	II	I							
		SZ31.1A	40 / 8	x			198	-46	4,5	II	I							
		SZ31.1B	40 / 8		X		246	-57	14,7	II	II							
		SZ31.1C	40 / 8			x												
58	SZ31	SZ31.2A	40 / 8	X			238	-52	1,8	II	I	4	213,2	-53,7	16,4	1	0	3,89
		SZ31.2B	40 / 8		X		180	-48	2,7	II	II							
		SZ31.3A	90 / 20	X			-	-	-	III	III							
		SZ31.3B	90 / 20		X		-	-	-	III	III							

Field Data				Analysis			ChRM directions			Sample Type		Site Mean Distribution					Watsons_f	
Height (m)	Sampling Site	Individual Sample	Dec / Inc	TH	AF	IRM	Dec	Inc	MAD	Visual	MAD	N	Dec	Inc	k	Class	F stats	Beat
59,5	SZ32	SZ32.1A	250 / 10	x			178	-30	4,9	II	II	6	175,7	-44,2	43,7	1	0	3,49
		SZ32.1B	250 / 10		X		186	-43	4	II	I							
		SZ32.1C	250 / 10			x												
		SZ32.2A	0 / 10	X			169	-51	1,6	II	I							
		SZ32.2B	0 / 10		X		181	-50	2,6	II	I							
		SZ32.3A	0 / 10	X			181	-45	4,7	II	II							
		SZ32.3B	0 / 10		X		187	-42	3,1	II	I							
61	SZ33	SZ33.1	90 / 10	x			7	-44	10,3	II	II	2	356	-55,4	18,4	1	0	6,94
		SZ33.2A	90 / 10		X		336,7	-65	2,6	II	I							
62,5	SZ0	SZ0.1	0 / 0	x			334	68	3,4	I	I	3	343,3	53,3	10,1	1	0	4,46
		SZ0.2	0 / 0		X		326	30	16,2	II	II							
		SZ0.3	0 / 0	X			17	55	7,3	II	II							
64	SZ1	SZ1.1A	0 / 0	x			349	61	8	I	II	4	355,8	45,3	11,6	1	0	3,89
		SZ1.1B	0 / 0		X		328	25	2,7	I	I							
		SZ1.2A	0 / 0	X			27	41	3,4	I	I							
		SZ1.2B	0 / 0		X		3	45	1,9	I	I							
		SZ1.3A	0 / 0			x												
65,5	SZ2	SZ2.1	0 / 0	x			12	15	2,5	I	I	2	11,5	13	774,8	1	0	6,94
		SZ2.2	0 / 0		X		11	11	4,1	I	I							
67	SZ3	SZ3.1	0 / 0	x			9	-26	5,3	I	II	1	9	-26	-	3		
		SZ3.2	0 / 0			x												
70	SZ5	SZ5.1	0 / 0	x			356	14	11,2	II	II	3	347,6	28,1	2,8	2	0	4,46
		SZ5.1bis	0 / 0		x		39	81	2,8	I	I							
		SZ5.2	0 / 0		X		332	-8,6	4,9	I	II							
		SZ5.4	0 / 0	x			-	-	-	III	III							
71,5	SZ6	SZ6.1	323 / 35	x			20	40	4,3	I	I	2	8,1	55,8	10,6	1	0	6,94
		SZ6.2	313 / 38		X		342	69	3,9	I	I							
		SZ6.4	329 / 32			x												
73	SZ7	SZ7.1A	0 / 0	x			18	28	6,9	I	II	6	366,6	36,8	3,1	2	0	3,49
		SZ7.1B	0 / 0		X		-	-	-	III	III							
		SZ7.2A	0 / 0	X			250	89	4,9	II	II							
		SZ7.2B	0 / 0		X		5	20	5,2	I	II							
		SZ7.3A	0 / 0	x			-	-	-	III	III							
		SZ7.3B	0 / 0		X		328	6	6,4	I	II							
		SZ7.4A	0 / 0	X			344	38	2,9	I	I							
		SZ7.4B	0 / 0		X		261	5	11,7	I	II							
		SZ7.4C	0 / 0			x												
74,5	SZ8	SZ8.1A	0 / 0	x			331	3	9,7	I	II	3	315	26,7	4,5	2	0	4,46
		SZ8.1B	0 / 0		X		265	52	3,9	I	I							
		SZ8.1C	0 / 0			x												
		SZ8.2A	0 / 0	X			-	-	-	III	III							
		SZ8.2B	0 / 0		X		327	18	5,8	I	II							

Figure 2

Reversal Test.

We have used a bootstrap test for a common mean to perform a reversal test (Tauxe, 1998), which is based on comparing the Cartesian coordinates of the bootstrapped means. This figure shows histograms of Cartesian coordinates of means of para-data sets drawn from the ChRM directions from Table I. The reversed polarity directions have been flipped to the antipode to test for a common mean of the two modes, normal and reversed. Because the confidence bounds from the two data sets overlap in all three components, the means of the reversed and normal modes cannot be distinguished at the 95% level of confidence. We therefore can conclude that the data set passes the bootstrap reversal test.

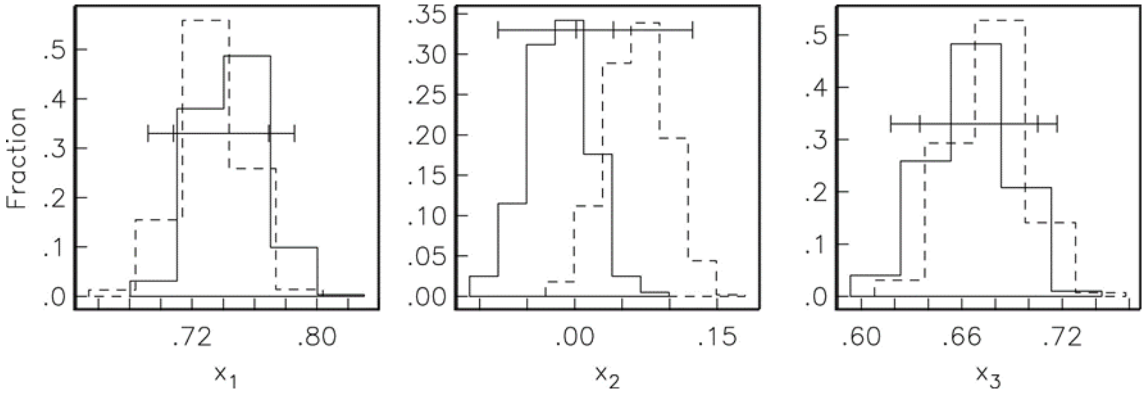

Supplement: Supplementary file 1 — Supplementary Information [file 41598_2017_14024_MOESM1_ESM.pdf]
